# Three-dimensional evaluation of the maxillary sinus in patients with different skeletal classes and cranio-maxillary relationships assessed with cone beam computed tomography

**DOI:** 10.1038/s41598-023-29391-5

**Published:** 2023-02-06

**Authors:** Andrea Abate, Davide Cavagnetto, Valentina Lanteri, Cinzia Maspero

**Affiliations:** 1 Department of Biomedical Surgical and Dental Sciences, University of Milan, 20142 Milan, Italy; 2 Fondazione IRCCS Cà Granda, Ospedale Maggiore Policlinico, 20100 Milan, Italy; 3 Department of Oral and Maxillofacial Surgery, Amsterdam University Medical Center (Amsterdam UMC), Location AMC, Meibergdreef 9, 1105 AZ Amsterdam, The Netherlands; 4 Politecnico of Turin, Turin, Italy

**Keywords:** Orthodontics, Three-dimensional imaging

## Abstract

The objective was to evaluate the relationship between the dimensions of the maxillary sinuses (MSs) and various cephalometric parameters. MS volume (MSV), MS surface (MSS), linear maximum depth (LMD), linear maximum width (LMW), and linear maximum height (LMH) were calculated on CBCT scans of 99 adults. Two sets of two-way (ANOVA) assessed the influence respectively of ANB and SNA angles and of the gender on MS dimensions. Pearson’s correlation was calculated between MS dimensions and different cephalometric variables. Reliability and accuracy of the proposed method was tested with intra-operator and inter-operator intraclass correlation coefficient (ICC). Two-way ANOVA showed no statistically significant difference in MSV, MSS and LMH between ANB groups, whilst males were associated with bigger sinuses. LMW showed statistically significant difference in both ANB and gender groups. LMD showed no statistically significant difference. The second Two-way ANOVA showed significantly larger MSV, MSS and LMD in patients with increased or reduced SNA angle but not between genders. LMW and LMH also showed a significant difference between genders. All linear measurements showed a significant interaction of the two factors. The intra-observer and inter-observer ICC scored high for all the tested measurements. MSV and MSS showed a positive correlation with S-N, PNS-A, S-Go, N-Me, N-Ans and the distance between Mx points. LMW had a negative correlation with Ba-S-N angle and N-Me, LMH with Ba-S-N angle, S-Go and Mx r-Mx l and LMD with N-Me and N-ANS. LMW had a positive correlation with Mx r-Mx l, LMH with S-N, S-N^Ans-Pns, N-Me, N-Ans and LMD with S-N, Ba-S-N, PNS-A, S-Go and distance between Mx points. In conclusion, MSV and MSS did not differ between the three skeletal classes, males showed significantly larger MS than in females. Concerning the influence of the cranio-maxillary relationship (SNA) and gender on MS dimension, subjects with a retrusion (SNA < 80°) or protusion (SNA > 84°) of the maxillary alveolar bone had larger MSV, MSS, LMW, LMH and LMD than subjects with a normal cranio-maxillary relationship (SNA 82 ± 2°). A statistically significant high positive correlation was observed between S-N, Pns-A, S-Go, Mx-R/Mx-r and MS dimension. Further studies that evaluate similar outcomes in different races may be able to enrich our knowledge on this topic.

## Introduction

Maxillary sinuses (MS) are the largest of the paranasal sinuses^1^. They are two bony chambers, located inside each maxillary bone. Being knowledgeable as to MS anatomy is a must, not only in forensics, but also in many dental and maxillofacial interventions.

The MS is the first paranasal sinus to form with growth starting during gestation, at the 12th week of pregnancy, as a lateral folding of the nasal epithelium^2^. Current literature^3,4^ reports that most of the MS postnatal growth occurs before the end of the 36th month, between seven and twelve years of age and adult size is reached between the ages of 12 and 15.

Maresh and Washburn^5^ were the first to assess posteroanterior teleradiographs and report observations and measurements of MS changes during adolescence. They emphasised that the growth of the MS varied according to the individual and varied widely^5^. Koymen et al.^6^ confirmed that MS dimension has individual variations and stated that facial biotype mat influence the morphology of the sinus. However, the data reported in international literature differ as to the growth and dimensions of the maxillary sinuses. This may be accounted for the fact that various methods have been applied for the evaluation of MS volume (MSV), including panoramic radiographs and lateral tele-radiographs, injection of various substances inside the MS and the application of the ellipsoid formula^7–9^.

However, advances in medical techniques have enhanced the evaluation of the maxillary sinuses. Indeed, various innovative diagnostic imaging techniques have been recently developed and have profoundly changed medical diagnostic capabilities. One such example is Cone Beam Computed Tomography (CBCT) in 1998^10^ that allows a low-dose accurate three dimensional assessment of craniofacial bony structures^11,12^.

Moreover, when CBCT scans are elaborated with dedicated 3D software, precise morphometric 3D measurements can be obtained for all the craniofacial structures^13^.

Current literature on maxillary sinuses is scant and several studies that are published rely on two-dimensional radiographs, such as orthopantomography (OPG) and cephalometric radiographs. However, these 2D radiographs provide limited information as the MS is a 3D structure and may well lead to erroneous conclusions.

Some recent studies have reported having made a 3D evaluation of the MS and the other paranasal sinuses and the rhynopharynx for surgical and medical purposes^9,14,15^. Others evaluated how the MS volume differs according to age and gender^16,17^. CBCT has certainly become today the most used method for the evaluation of maxillary sinuses, highlighting a high reliability of the method as reported by different authors^18–20^.The literature searches in Pubmed, Scopus, EbSco performed by the authors revealed a limited number of papers investigating the relationship between MS size and cephalometric characteristics. Moreover, the only ones were performed on two-dimensional radiographs.

Oktay^1^ stated that the dimension of the MS was not influenced by malocclusion nor gender, and that only in Angle Class II, sex was a significant component.

Basdra et al.^21^ made a thorough evaluation of a subject with an asymmetric lower jaw due to changes in MS surfaces and reported that the MS may have an impact on facial growth.

The most recent study on the relationship between MS and malocclusion, has been proposed by Endo et al.^22^, who assessed MS dimensions and their relationship with Angle's dental classes and several cephalometric measurements using bidimensional radiographs.

Therefore, the aim of the present study is to provide a 3D assessment of the maxillary sinuses, by investigating volume (MSV), surface (MSS) and linear maximum height (LMH), width (LMW) and depth (LMD) and to perform a comparison between male and female subjects in the three different skeletal classes and cranio-maxillary relationship and to assess the relationship between the linear and volumetric measurements of the MS and the patients’ craniofacial characteristics.

## Methods

The null hypothesis states that there is no difference in the maxillary sinus dimensions in male and female subjects having different skeletal classes (based on ANB) and cranio-maxillary relationships (based on SNA).

The CBCT scans of 99 MS of Caucasian patients were retrospectively examined at the Department of Biomedical Surgical and Dental Sciences, University of Milan, 20142 Milan, Italy.

The study presented herein, was approved by the Ethical Committee of Fondazione IRCCS Ca’Granda, Ospedale Maggiore, Milan—Italy (protocol n.573/15). All patients gave written informed consent for the use of medical records in anonymous form for research purposes.

### Participants and inclusion criteria

This cross-sectional study was carried out by the assessment of cone beam computed tomographies taken between 2009 and 2019 at the Department of Radiology of Fondazione IRCCS Ca’Granda, Ospedale Maggiore, Milan. The patients were selected from the archives according to the following inclusion criteria: being Caucasian; nasal breathers; having all permanent teeth erupted apart from wisdom teeth; patients 16 years of age or older so as to evaluate subjects who were no longer or only slightly growing at the level of the maxillary sinus^22,23^.

The Exclusion criteria were: present or past pathological involvement of the maxillary sinuses; missing maxillary molars or premolars; previous orthodontic treatment; altered bone metabolism; skeletal asymmetry; alterations to the maxillofacial skeleton (acquired or congenital); mouth breathers.

A thousand five hundred fifty records were reviewed. On the basis of the exclusion/inclusion criteria, 99 Cone Beam Computed Tomographies of White subjects (47 males and 52 females), aged 20.9 ± 2.1 years, were selected for the study and categorized into three groups, according to anteroposterior skeletal classes based on Riedel’s Analysis ANB angle^24^: skeletal groups Class I (ANB 2 ± 2°), 34 subjects, Class II (ANB > 4°), 31 subjects, and Class III (ANB < 0°), 34 subjects. The patients were also categorized into 1 of the 3 cranio-maxillary relationships determined by the horizontal prognathism of the maxillary alveolar bone, based on Downs’ SNA angle^25^: Normatrusive (SNA 82 ± 2°) 35 subjects, protrusive (SNA > 84°) 33, retrusive (SNA < 80°) 31 subjects.

After assessing the absence of any asymmetries between the right and left MS in the sample, the average between the two sides was calculated and used for simplicity throughout the study. The following parameters were evaluated: MS volume (MSV), MS surface (MSS), linear maximum width (LMW), linear maximum depth (LMD) and linear maximum height (LMH). All the parameters were calculated by Mimics ResearchTM software version 21.0 (NV, Technologielaan 15, 3001 Leuven, Belgium). A cephalometric tracing has been performed for each subject to evaluate the association between craniofacial features and maxillary sinus characteristics.

### CBCT examination and data processing

All patients received a CBCT scan using the same machine, I-CAT FLX (Imaging Sciences International, Hatfield, PA, USA). The scanning parameters were configured as follows: a 360° rotation, 300 frames, 120 kV[p], 5 mA, 3.7 s, a voxel size of 0.3 mm and one of the three field of view (FOV) 20 mm × 17 mm, 16 mm × 8 mm and 16 mm × 11 mm. The field of view was tailored for each patient to minimize radiation exposure, with a distance between the 2 slices of 0.3 mm to ensure anatomic registration accuracy. The scans were taken by one expert operator that used three position aids to correctly place the patient into natural head position and registered in the DICOM (Digital Imaging and Communications in Medicine)format. The CBCT scans were then loaded into Mimics Research™ v.16 software (NV, Technologielaan 15, 3001 Leuven, Belgium, https://www.materialise.com/en/medical/mimics-innovation-suite/mimics) to perform all the assessments.

**Figure 1:**
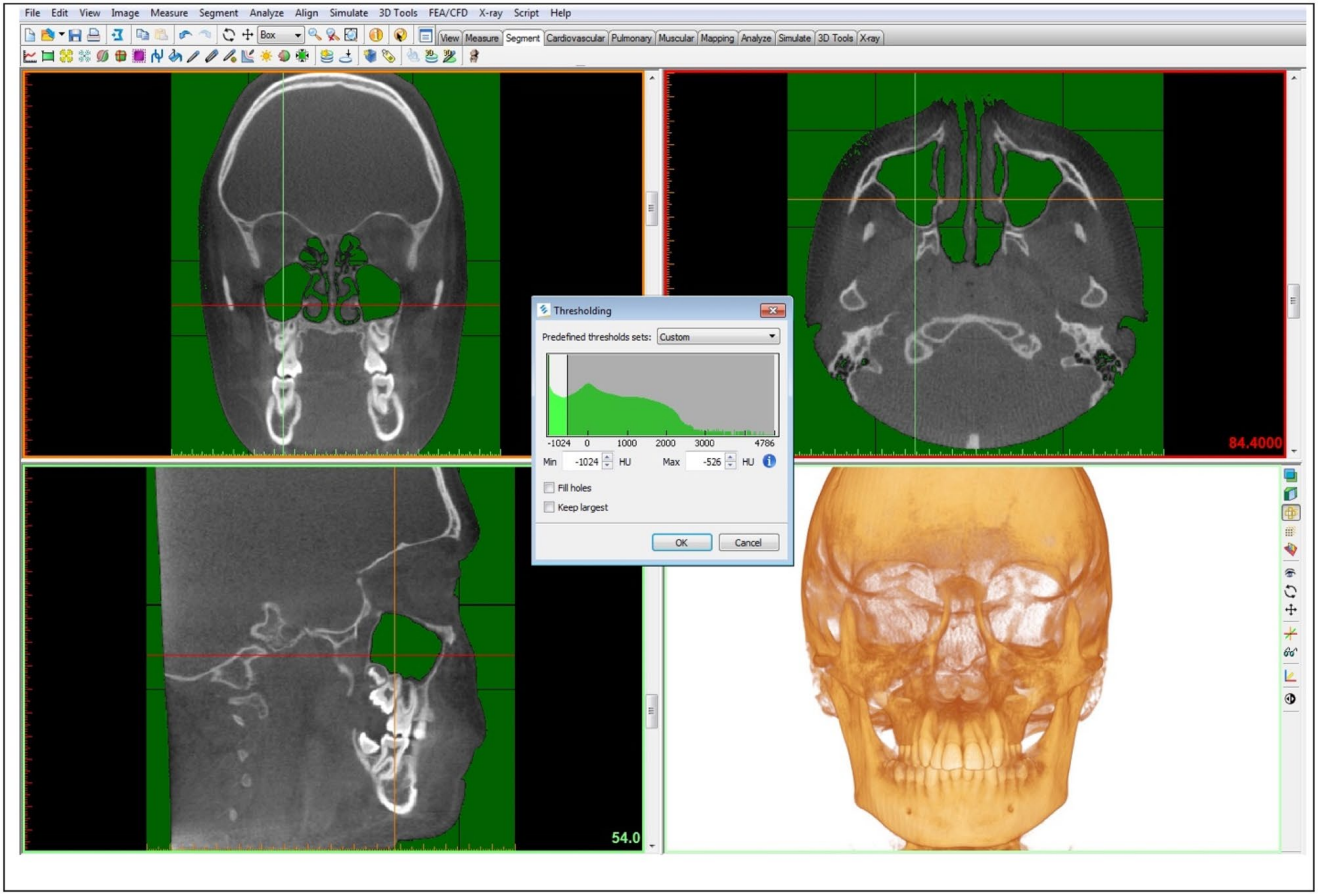
Thresholding function of the Mimics Research™ v.16 software (NV, Technologielaan 15, 3001 Leuven, Belgium, https://www.materialise.com/en/medical/mimics-innovation-suite/mimics) was used to segment each MS: from − 1024 HU (minimum) to − 526 HU.

A specific threshold was set for each MSV calculation. The threshold limits were from a minimum of − 1024 HU (minimum) to a maximum of − 526 HU (Fig. 1), as reported by Sahlstrand-Johnson et al.^26^.

**Figure 2:**
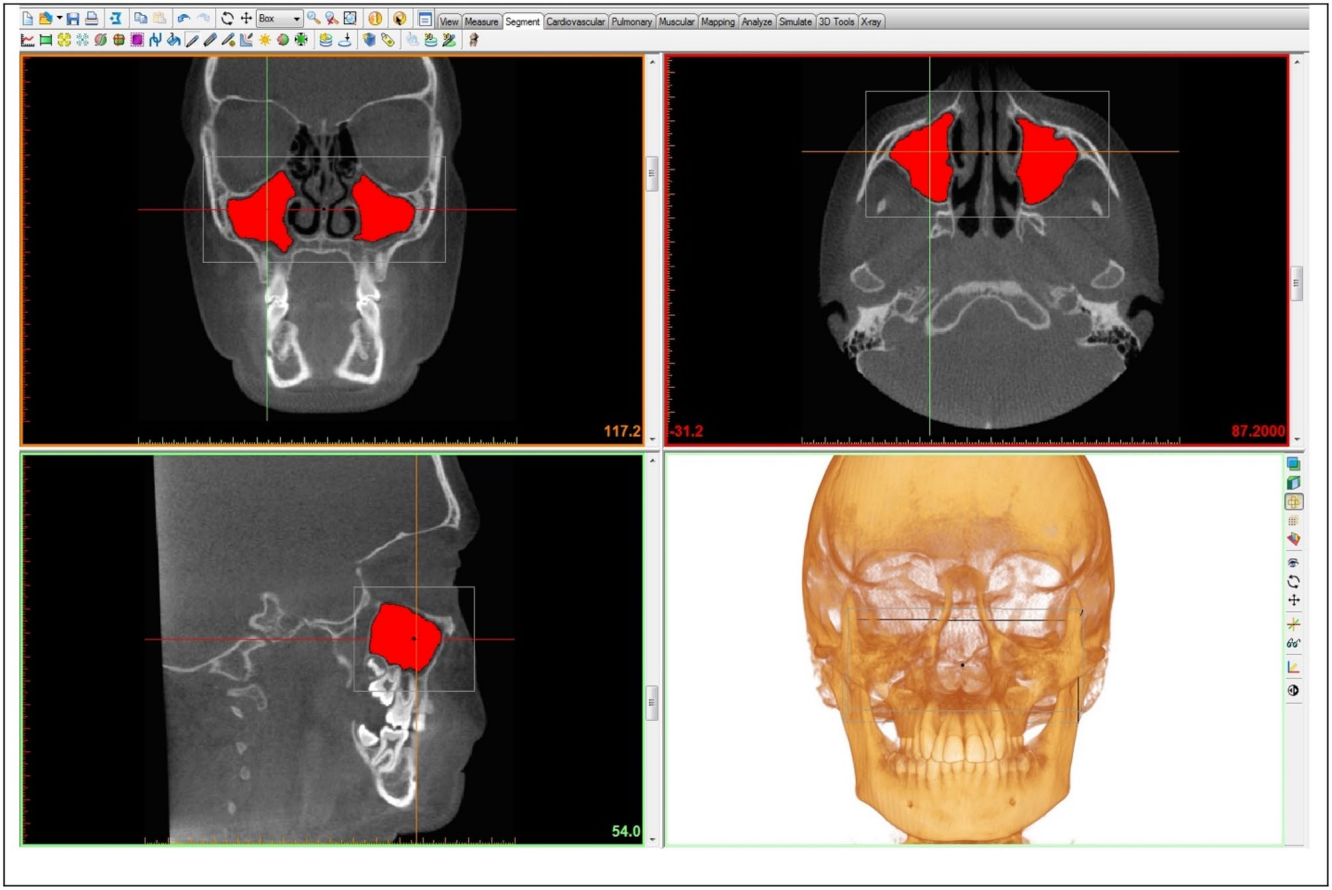
The region growing tool was used to separate and isolate MSs using Mimics Research™ v.16 software (NV, Technologielaan 15, 3001 Leuven, Belgium, https://www.materialise.com/en/medical/mimics-innovation-suite/mimics).

**Figure 3:**
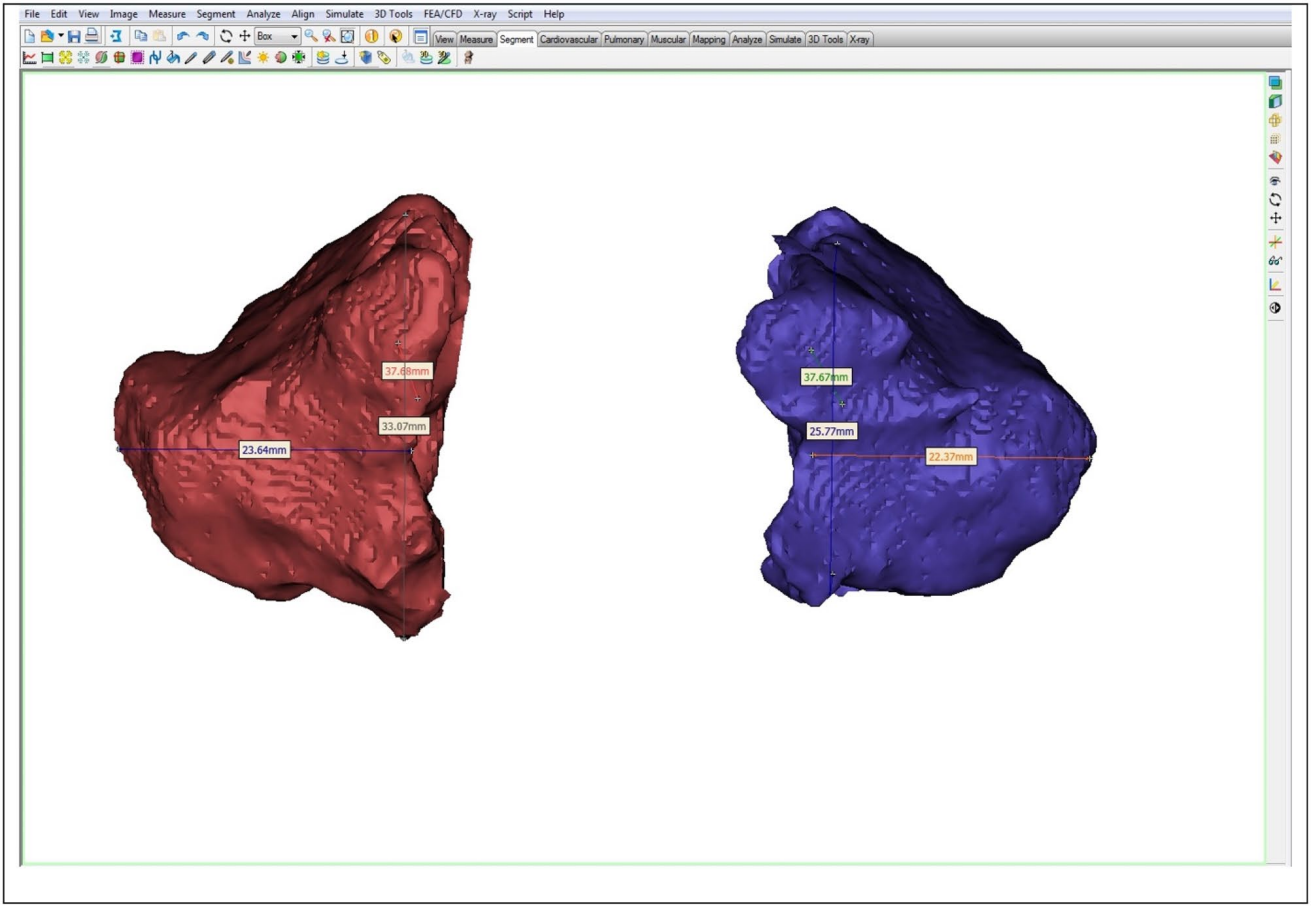
Measurements of the MSs automatically generated by Mimics Research™ v.16 software (NV, Technologielaan 15, 3001 Leuven, Belgium, https://www.materialise.com/en/medical/mimics-innovation-suite/mimics).

Using a software tool of Mimics Research known as, “edit masks,” according to a procedure already validated by Maspero et al.^16^ and Motro and Erverdi^27^, each MS was cropped along the borders, and at the level of the smallest tract of the *hiatus* between the processus uncinatus and the infundibulum. The segmentation tools of Materialise Mimics were used to crop the *hiatus* of the MS slice by slice (Fig. 2). The function “calculate 3D” was used to calculate MSV^16,27^ (Fig. 3).

After segmentation, the 3D volumetric structure of the MSV (right and left) of each patient was calculated separately. Flood-fill and smoothing operations were applied to the airway mask to calculate the total volume, whatever the porosity in the MSV. The volume and surface of the selected structures was then calculated by the software Mimics™. Lastly, the LMW, the LMH and the LMD parameters were automatically calculated by the Mimics Research™ software.

**Figure 4:**
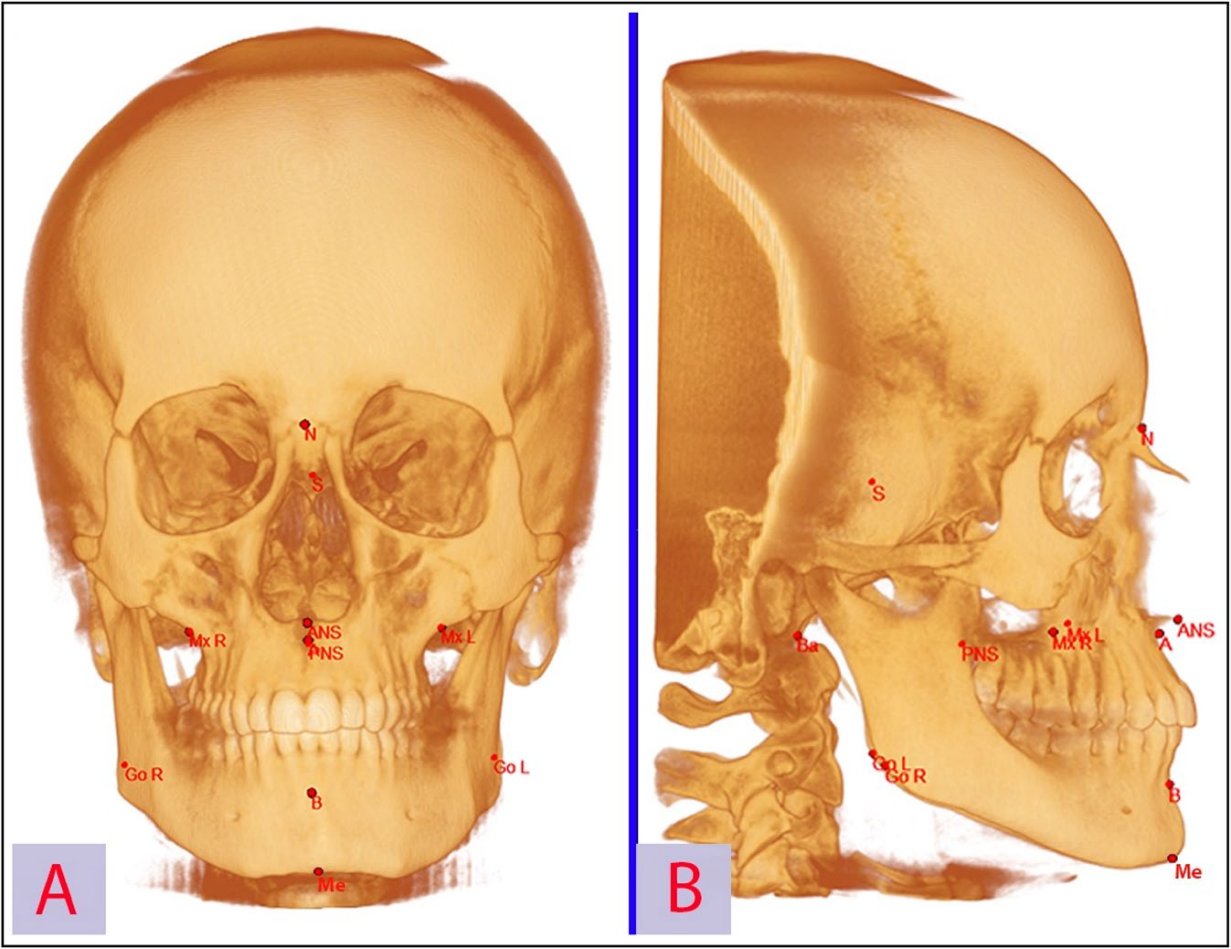
Cephalometric landmarks used in the present study were: S = Sella (centre of sella turcica); N = Nasion (most anterior limit of the frontonasal suture on the frontal bone in the facial midline); Ba = Basion (The most anterior point of the foramen magnum); ANS = Anterior nasal spine (the tip of the anterior nasal spine); PNS = Posterior nasal spine (the most posterior point on the bony hard palate); A = Point A of Downs (Deepest bony point on the contour of the premaxilla below ANS); B = Point B of Downs (Deepest bony point of the contour of the mandible above pogonion); Me = Menton (most inferior point of mandibular symphysis); GO l/r = left/right Gonion points (located at the most posterior inferior point of the gonial angle of the mandible); MX l/r = left/right maxillare (the deepest point of the concavity formed by the lateral wall of the maxilla and the inferior border of the zygomatic process of the maxilla).

As regard to the correlation analysis, 11 craniofacial measurements (6 linear and 5 angular) were traced on the CBCT scans, using Mimics Research™ software, to evaluate their relationship with the MSV, MSS and LMW, LMH and the LMD. Figure 4 summarized the cephalometric points used in the present study. The cephalometric measurements used in this study are reported in Table 1.

**Table 1:** Definition of the cephalometric measurements used in the present study.

Linear measurements	Angular measurement
*Maxillary length (PNS-A)*: the distance between the posterior nasal spine (PNS) and point A	*SNA*: the angle formed between points S, N, and A, indicating the anteroposterior projection of the maxilla
*Anterior cranial fossa length (S-N)*: the distance between sella (S) and nasion (N)	*SNB*: the angle formed between points S, N, and B, indicating the anteroposterior projection of the mandible
*Total anterior facial height (N-Me)*: the distance between nasion (N) and menton (Me)	*ANB*: the angle formed between points A, N, and B, indicating the anteroposterior intermaxillary relationship. In 3D analysis, unlike traditional cephalometrics, the difference between SNA and SNB could differ from the value of ANB
*Upper anterior facial height (N-ANS)*: the distance between nasion (N) and the anterior nasal spine (ANS)	*Cranial base angle (Ba-S-N)*: the angle between basion (Ba), S, and N
*Posterior facial height (S-Go L/R)*: the distance between sella (S) and left and right Gonion (Go)	*Craniomaxillary angle (SN-PNS-ANS)*: the angle between the floor of the anterior cranial fossa and the palatal plane
*Maxillary width (Mx R-Mx L)* the distances between the right and left maxillary points	

### Statistical analysis

A preliminary analysis of the sample size power was carried out using G*Power (version 3.1.9, http://www.psychologie.hhu.de/arbeitsgruppen/allgemeine-psychologie-und-arbeitspsychologie/gpower.html) on 30 subjects (10 for each skeletal class); power calculation analysis was performed using the average values of the MSV for each skeletal class, the number of subjects analyzed for each group and the common standard deviation. The following data were used to calculate sample size: MSV skeletal class I = 13,339, skeletal class II = 12,632, skeletal class III = 14,924; group size = 10; σ within each group = 2460; α = 0.05; with a beta error level of 20%. Sixty-nine subjects (23 in each group) would have provided 80% power. However, according to the inclusion criteria, the authors could include 31 patients per group, thus increasing the robustness of the data. Statistics were carried out by the software SPSS v. 25.0 (IBM, Chicago, IL, USA). The Kolmogorov–Smirnov test was used to assess whether the data was normally distributed if the dataset was greater or equal to 50. It was observed that the statistical distribution of the quantitative measurements was Gaussian. Each measurement was reported as mean and standard deviation. The dimensional variations between the right and left MSV, MSS, LMW, LMH and LMD of the MS in the male and female group were analyzed. Statistically significant differences between the left and right sides in each measurement were computed by paired Student's t test.

After ensuring that the right and the left maxillary sinuses were not statistically significant different, the average values between the two sides were used for the following statistical analysis. After testing necessary assumptions, normality assessment in all the subgroups of each variable (via Shapiro–Wilk test) and homogeneity of the variances of the dependent variables in all the evaluated subgroups (via Levene’s statistics), a two-way analysis of variance (ANOVA) was used to test the influence of two different categorical independent variables, which were gender and skeletal malocclusion, on one dependent variable represented by MSV, MSS, LMW, LMH and LMD. The ANOVA was also calculated to assess the influence of gender and maxillary prognathism had on MSV, MSS, LMW, LMH and LMD.

The ANOVA test was also used to assess whether there was any interaction between the two-independent variables. The post hoc Sheffe’s test was used for within-group comparisons. Pearson’s correlation analysis was computed to investigate for any association between MSV, MSS, LMW, LMH and LMD and craniofacial morphological measurements. Intra-class correlation coefficient (ICC) was used to assess intra-examiner reliability the whilst the Dahlberg’s formula to measure the method error^28^.

Concerning the method error, all the maxillary and cephalometric measurements were obtained by a single expert orthodontist (A.A.) specialized in 3D radiologic imaging. After two weeks, 30 randomly selected CBCT were evaluated by a different expert orthodontist (D.C.), and then recalculated by A. A. to assess the intra-observer and inter-observer reliability. Both A.A. and D.C. were blinded to the patients’ identity and processed one scan per day to prevent fatigue errors.

Statistical significance was defined at a p-value < 0.05.

### Ethical approval and consent to participate

The study protocol was approved by the Ethical Committee of the Fondazione IRCCS Ca’Granda, Ospedale Maggiore, Milan—Italy (protocol n.573/15). All procedures performed in this retrospective study involving human participants were in accordance with the ethical standards of the institutional and/or research committee and with the 1964 Helsinki declaration and its later amendments or comparable ethical standards.

### Informed consent

For this type of study, informed consent was obtained from all parents’ patients or their guardians.

## Results

Mean, standard deviation of the considered variables (MSV, MSS, LMH, LMW and LMD) and their comparison using paired t-test between the two sides and independent t test between gender are reported in Table 2. MSV, MSS, LMW, LMH and LMD did not show any statistically meaningful difference between the right and left side in the male and female groups. Whilst there was a statistically meaningful difference between genders for each variable evaluated. (Table 2).

**Table 2:** Descriptive statistics for MS measurements; paired student T test between right and left side to assess symmetry.

Measurement	Group	Male		p value	Female		p value	(Male vs female)
		Mean (mm^3^)	SD		Mean (mm^3^)	SD		P value
MSV	Right	13,500.371	2826.307	0.143	12,254.814	1915.694	0.152	0.034
	Left	13,976.564	3004.764		11,752.872	1793.563		0.021
MSS	Right	4229.044	1221.197	0.092	3499.825	480.104	0.296	0.014
	Left	3974.757	976.432		3867.435	653.277		0.026
LMW(X)	Right	32.733	3.564	0.167	29.448	3.039	0.091	0.011
	Left	29.768	2.983		27.734	2.335		0.038
LMH (Y)	Right	41.635	4.0141	0.276	34.126	2.228	0.395	0.009
	Left	38.843	3.124		37.217	1.984		0.045
LMD (Z)	Right	34.412	4.628	0.346	34.018	3.695	0.267	0.041
	Left	38.249	3.933		37.124	3.016		0.033

Independent t-test was used to compare male and female subjects. A p value < 0.05 was considered statistically significant.

### The influence of skeletal class (ANB) and gender on MS dimension

Table 3 reports the results as to the influence the anteroposterior skeletal class ANB and gender had on the maxillary sinus.

**Table 3:** Two-way ANOVA with ANB and gender as categorical independent variables and MS measurements.

Measurement	n	Male		n	Female		Two-way anova				
							Source	F	Partial eta squared	p value	Post-hoc analysis with Sheffe’ adjustment
		Mean (mm^3^)	SD		Mean (mm^3^)	SD					
Maxillary sinus volume (MSV)—(mm^3^)											
Class II	14	14,339.784	2372.273	14	12,667.932	1263.224	Skeletal classes	1.396	0.031	0.251	–
Class I	14	12,632.675	2147.625	20	12,144.421	1604.979	Sex	10.004	0.104	**0.026**	M > F
Class III	19	14,264.468	2948.071	18	11,531.238	2247.403	Interaction	1.981	0.044	0.143	–
Maxillary sinus surface (MSS—(mm^2^)											
Class II	14	4096.043	597.968	14	3854.094	205.931	Skeletal classes	0.148	0.003	0.868	–
Class I	14	3904.464	847.915	20	3785.797	456.635	Sex	4.030	0.045	**0.046**	M > F
Class III	19	4258.717	654.203	18	3496.248	732.368	Interaction	1.375	0.031	0.269	–
Linear maximum depth (LMD)—(mm)											
Class II	14	26.673	1.402	14	25.802	0.523	Skeletal classes	5.003	0.104	**0.012**	2 < 1; 2 < 3; 3 < 1 *(Fig. 5)
Class I	14	31.825	8.786	20	30.591	2.068	Sex	27.957	0.245	< 0.01	M > F
Class III	19	28.887	3.748	18	28.487	2.359	Interaction	12.387	0.224	0.231	–
Linear maximum depth (LMD)—(mm)											
Class II	14	40.453	1.745	14	35.663	0.499	Skeletal classes	1.818	0.041	0.172	–
Class I	14	34.495	4.633	20	35.905	2.554	Sex	21.227	0.198	** < 0.01**	M > F
Class III	19	38.751	3.117	18	36.698	2.073	Interaction	2.208	0.049	0.126	–
Linear maximum depth (LMD)—(mm)											
Class II	14	36.667	4.047	14	38.423	0.422	Skeletal classes	2.058	0.046	0.133	–
Class I	14	36.055	2.648	20	35.356	0.915	Sex	0.052	0.001	0.827	–
Class III	19	36.363	5.484	18	34.748	4.814	Interaction	1.343	0.030	0.275	–

Significant values are in bold.

A p value < 0.05 was considered statistically significant.

The ANOVA test showed did not find any statistically significant result between the MSV and MSS in the three skeletal classes (ANB) group. A significant difference (p-value < 0.05) was observed between males and females for the same variables.

**Figure 5:**
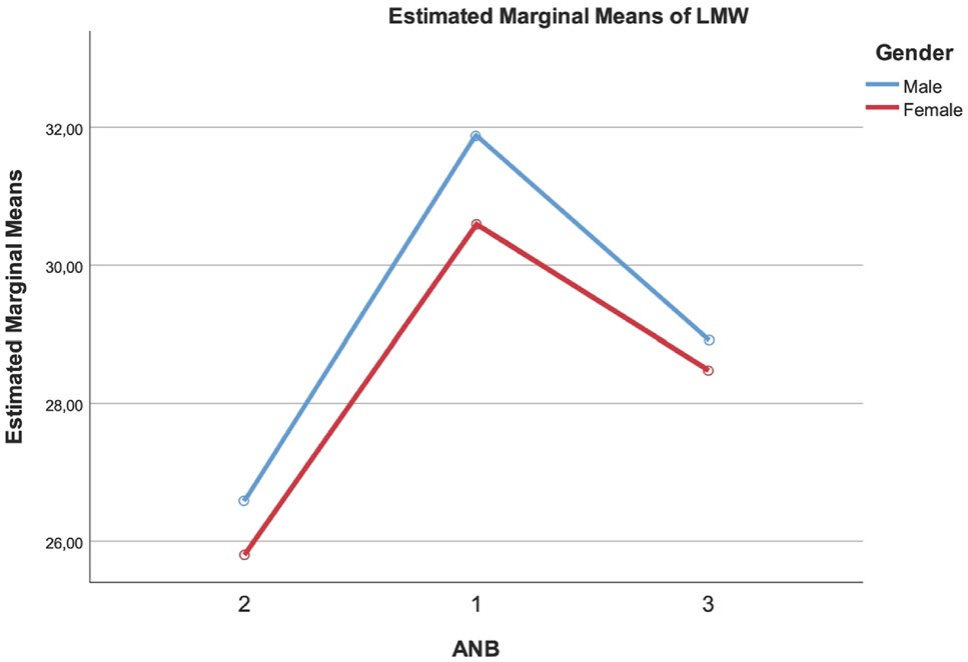
The plot of the mean LMW measurements for each combination of groups of Gender and ANB are plotted in a line graph.

There was a statistically significant difference in LMW (p-value < 0.05) between the three skeletal class groups and between males and females. Skeletal classes I had a bigger LMW than class III and class II, the latter showed a shorter LMW than skeletal class III (Fig. 5).

Although no statistically significant difference was observed for LMH in the three skeletal classes, there was a significantly bigger LMH in the male subjects (p-value < 0.05). There was no statistically significant difference in the LMD in the three skeletal classes and between genders.

### The influence the cranio-maxillary relationship (SNA) and gender have on MS dimension

Table 4 reports the results of the influence the cranio-maxillary relationship (SNA) and gender have on the maxillary sinus. The ANOVA test evidenced a statistically significant difference (p-value < 0.05) between the MSV and MSS in the three horizontal prognathism of the maxillary alveolar bone (SNA) group. Patients with retrusive (SNA < 80°) and protrusive (SNA > 84°) maxilla had a larger MSV and surface than did subjects with normal cranio-maxillary relationships (SNA 82 ± 2°).

**Table 4:** Two-way ANOVA with SNA and gender as categorical independent variables and MS measurements.

Measurements	n	Male			Female		Two-way anova				
							Source	F	Partial eta squared	p value	Post-hoc analysis with Sheffe’ adjustment
		Mean	SD	n	Mean	SD					
Maxillary sinus volume (MSV)—(mm^3^)											
Retrusive (R)	17	15,105.194	2475.462	17	12,667.937	1263.225	SNA classes	11.935	0.217	** < 0.01**	R > N; P > N
Normotrusive (N)	14	11,472.906	1814.773	21	11,235.508	1503.247	Sex	2.762	0.031	0.105	–
Protrusive (P)	16	13,348.557	2718.706	14	13,644.783	296.373	Interaction	3.050	0.066	0.083	–
Maxillary sinus surface (MSS)—(mm^2^)											
Retrusive (R)	17	4570.893	1293.987	17	3854.096	205.932	SNA classes	7.684	0.152	**0.015**	R > N; P > N
Normotrusive (N)	14	3397.886	528.326	21	3475.848	622.790	Sex	0.596	0.007	0.443	–
Protrusive (P)	16	3906.902	698.402	14	4136.534	228.154	Interaction	2.755	0.060	0.071	–
Linear maximum width (LMW)—(mm)											
Retrusive (R)	17	32.941	3.396	17	25.803	0.525	SNA classes	4.595	0.097	**0.018**	R > N; P > N
Normotrusive (N)	14	29.587	2.319	21	28.360	2.151	Sex	14.301	0.143	** < 0.01**	M > F
Protrusive (P)	16	29.813	2.371	14	32.063	1.387	Interaction	24.693	0.365	** < 0.01**	*(Fig. 6)
Linear maximum height (LMH)—(mm)											
Retrusive (R)	17	39.744	2.965	17	36.661	0.485	SNA classes	11.440	0.210	** < 0.01**	R > N; P > N
Normotrusive (N)	14	35.706	4,201	21	35.608	2.301	Sex	9.647	0.101	** < 0.01**	M > F
Protrusive (P)	16	39,581	2.595	14	38.384	0.226	Interaction	4.506	0.095	**0.013**	*(Fig. 6)
Linear maximum depth (LMD)—(mm)											
Retrusive (R)	17	38,115	3,155	17	38.423	0.423	SNA classes	17.444	0.289	** < 0.01**	R > N; P > N
Normotrusive (N)	14	31,670	3,241	21	34.795	3.931	Sex	0.813	0.009	**0.376**	–
Protrusive (P)	16	37,213	4,017	14	35.818	0.637	Interaction	3.209	0.069	**0.045**	*(Fig. 6)

Significant values are in bold.

A p value < 0.05 was considered statistically significant.

Males and females did not present any statistically significant difference for the same variables.

**Figure 6:**
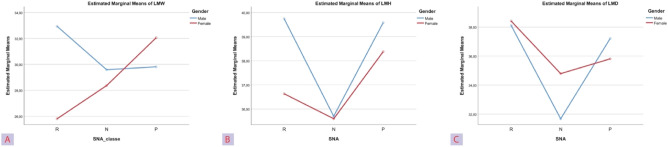
The plots of respectively the mean LMW (**A**), LMH (**B**), LMD (**C**) measurements for each combination of groups of Gender and SNA are plotted in a line graph.

The LMW and LMH values were superimposable. There was a statistically significant difference (p-value < 0.05) in both these variables between the three cranio-maxillary relationship groups. Patients with retrusive (SNA < 80°) and protrusive (SNA > 84°) maxilla had a significant increase of the LMW and LMH (p-value < 0.05) than subjects with normal cranio-maxillary relationships (SNA 82 ± 2°). For the same variables a statistically significant differences were observed between males and females (Table 4). Moreover, the ANOVA test evidenced a statistically significant interaction (p-value < 0.05) between the two independent variables (SNA; Gender; see Fig. 6a,b). There was a statistically significant difference in linear maximum depth (LMD) (p-value < 0.05) between the three SNA group. Both the retrusive and protrusive group had significantly larger LMD (p-value < 0.05) than patients with normal SNA (Table 4). No significant difference was observed between gender for the same variable. A significant interaction (p-value < 0.05) between the two independent variables (SNA; Gender) was also noted (Fig. 6c).

The simple main effect analysis revealed the different behavior of LMW, LMH and LMD in the SNA classes among gender groups (see Table 5). In males LMW was significantly higher in the retrusive group compared to normal and to protrusive groups, LMH and LMD were significantly lower in the normal group compared to the retrusive and to the protrusive groups. In females LMW was greater in the normal group compared to the retrusive group and was greater in the protrusive group compared to normal and to retrusive group. LMH was higher in the protrusive and normotrusive groups. LMD was greater in the retrusive group compared to normal group. The simple main effect analysis revealed the different behavior of LMW, LMH and LMD in the gender groups among SNA classes (see Table 6). In the retrusive group of LMW and LMH males showed statistically significant higher values compared to female subjects whereas the opposite occurred in the normotrusive group of LMD variable.

**Table 5:** Simple main effects: Effect of gender on MS measurements at each level of SNA angle.

	Gender	P value	Pairwise comparison				
			Couples		Mean difference	Std. error	p value
LMW	Male (n = 47)	** < 0.001**	R	N	3.352	0.918	** < 0.001**
			R	P	3.129	0.866	**0.001**
			N	P	− 0.223	1.015	0.827
	Female (n = 52)	** < 0.001**	R	N	− 2.563	0.866	**0.004**
			R	P	− 6.255	1.060	** < 0.001**
			N	P	− 3.692	0,866	** < 0.001**
LMH	Male (n = 47)	** < 0.001**	R	N	4.045	0.985	** < 0.001**
			R	P	0.160	0.929	0.863
			N	P	− 3.885	1.089	**0.001**
	Female (n = 52)	**0.011**	R	N	0.065	0.929	0.944
			R	P	− 2.720	1.137	**0.019**
			N	P	− 2.785	0.929	**0.004**
LMD	Male (n = 47)	** < 0.001**	R	N	6.438	1.262	** < 0.001**
			R	P	0.898	1.190	0.453
			N	P	− 5.540	1.395	** < 0.001**
	Female (n = 52)	**0.012**	R	N	3.625	1.190	**0.003**
			R	P	2.605	1.457	0.077
			N	P	− 1.020	1.190	0.394

Significant values are in bold.

A p value < 0.05 was considered statistically significant.

**Table 6:** Simple main effects: Effect of different maxillary positions (SNA angles) on MS measurements at each level of gender.

	SNA_classe	Comparison couples		Mean difference	Std. error	p value
LMW	R	M (n = 17)	F (n = 17)	7.141	0.918	** < 0.001**
	N	M (n = 14)	F (n = 21)	1.226	0.866	0.160
	P	M (n = 16)	F (n = 14)	− 2.243	1.015	**0.030**
LMH	R	M (n = 17)	F (n = 17)	4.086	0.985	** < 0.001**
	N	M (n = 14)	F (n = 21)	0.105	0.929	0.910
	P	M (n = 16)	F (n = 14)	1.205	1.089	0.271
LMD	R	M (n = 17)	F (n = 17)	− 0.301	1.262	0.812
	N	M (n = 14)	F (n = 21)	− 3.113	1.190	**0.011**
	P	M (n = 16)	F (n = 14)	1.407	1.395	0.316

Significant values are in bold.

A p value < 0.05 was considered statistically significant.

### The correlation analysis between cephalometric values and MS dimension

Pearson’s correlation coefficients between the dimensions of the MS and craniofacial cephalometric variables are reported in Table 7. There was a medium to high positive correlation with S-N, PNS-A, Mx r-Mx l, S-Go, N-Me and N-Ans variables. MSS evidenced a significant positive correlation with S-N, Pns-A and Mx r-Mx l, N-Me and N-Ans variables. A significant negative correlation was observed between the LMW, Ba-S^-N and N-Me and there was a statistically significant positive correlation with Mx r-Mx L. There was a significant positive correlation in LMH for the following variables: S-N, S-N-Ans-Pns, N-Me, N-Ans. Whilst there was a significant negative correlation between LMH and Ba-S-N, S-Go and Mx r-Mx l. A significant positive correlation was observed between the LMD and S-N, Ba-S-N, PNS-A, S-Go and Mx r-Mx l. Furthermore, LMD was negatively correlated to N-Me and N-Ans.

**Table 7:** Pearson’s correlation coefficients between MS measurements and cephalometric variables taken into consideration.

	MSV	MSS	LMW	LMH	LMD
Cranial base					
S-N (mm)	0.380^‡^	0.156^†^	0.100	0.148†	0.290^‡^
Ba-S-N (°)	− 0.143	− 0.008	− 0.229^†^	− 0.284^‡^	0.296^‡^
Maxilla and mandible					
SNA (°)	− 0.122	− 0.157	− 0.014	0.069	− 0.126
SNB (°)	− 0.225	0.261	− 0.091	0.036	− 0.163
ANB (°)	0.034	0.015	− 0.106	− 0.019	0.098
PNS-A (mm)	0.432^‡^	0.267^‡^	0.342	0.028	0.235^‡^
S-N-Ans-Pns (°)	0.108	0.028	− 0.175	0.226^†^	0.160
Vertical dimension					
N-Me (mm)	0.455^‡^	0.655^‡^	− 0.388^‡^	0.387^‡^	− 0.239^†^
S-go (mm)	0.231^†^	− 0.089	0.138	− 0.246^†^	0.466^‡^
N-Ans (mm)	0.379^‡^	0.514^‡^	− 0.073	0.266^†^	− 0.266^†^
Transverse dimension					
Mx R-Mx L (mm)	0.430^‡^	0.352^‡^	0.595^‡^	− 0.370^‡^	0.265^†^

A p value < 0.05 was considered statistically significant (^†^p < 0.05; ^‡^p < 0.01).

The average intra-observer and inter-observer ICC (average ± SD, range) scored high: 0.975 ± 0.012, 0.946–0.993 and 0.963 ± 0.016, 0.934–0.982 respectively. According to Dahlberg’s formula, the random error for sinus volumetric and linear measurements was 446 mm^3^ for the MSV, 112 mm^3^ for the MSS and about 0.67 mm for the linear measurements. The random error for cephalometric measurements was about 0.48 mm for linear measurements and 0.23° for the angular.

## Discussion

Various papers have looked into MS morphometry and MS volumetric changes in relation to several conditions, i.e., different orthodontic appliances, nasal septum deviation, and sinus pathologies^29–31^. Koppe et al.^32^ took into consideration the MSV and the characteristics of the maxilla-facial skeleton of adults with an untreated bilateral cleft and negative control patients and reported that bigger skulls had greater MSs and that cleft patients had larger paranasal sinuses than the control subjects. On the contrary, Erdur et al.^33^ found that MSs of unilateral cleft patients had no statistically significant difference between sides. Other studies also evaluated the relationship between MS size with gender and age^29–31^.

Nevertheless, to the best of authors’ knowledge, the present study is the first human CBCT investigation to make a 3D analysis of the volume, surface and linear maximum width depth and height of the MS and to compare these data between three different skeletal classes, evaluating the relationship between the shape of the maxillary sinuses and the patients’ craniofacial features in adult subjects. Bidimensional orthopantomography and lateral cephalogram presents several shortcomings in assessing three dimensional structures as already investigated in literature. Every clinician with some experience with OPG knows that even in the same patient the surface of the maxillary sinuses may vary greatly because it is strictly dependent on the inclination of the head to the x-ray beam that causes different projection on the 2D sensor of two pyramid shape volumes with a particular orientation inside the maxilla.

All papers but one investigated the surface of maxillary sinuses on bidimensional radiographs therefore providing inaccurate data and making their conclusions almost worthless.

The only other study^34^ that investigated the three-dimensional volume of maxillary sinuses and its correlation to cephalometric indexes was published last year. Our study aimed at giving more information on correlations between cephalometric indexes and maxillary sinuses dimensions taking into account gender differences as it was not considered in the previous publication.

The data obtained in this study evidenced no significant difference for MSV, MSS, LMW, LMH and LMD between the right and left side, demonstrating the absence of asymmetry in the MS in the present sample.

A statistically significant difference was registered between genders for the MS with males having a larger sinus than females for each variable evaluated. One paper^35^ described that the maxillary sinus volume continued to grow until 20 years of age and then began reducing its size and their findings were in line with those in this study, in as much as they reported no significant difference between the right and left MSVs (Table 2).

The data of the present study showed no significant difference between the three skeletal class (ANB) group for the MSV and surface, evidencing no association between these variables and the anteroposterior relationship. There was a larger volume and surface in males than in females, in contrast with previously published literature data, i.e., Endo et al.^22^ reported having observed no statically significant differences in the maxillary morphological measurements between genders. Oktay’s study used the Duncan’s multiple comparison test to determine the size of maxillary sinuses and reported that that Class I female subjects and Class II and III male subjects had smaller MS than Class II female subjects^1^. The differences in these two results may be attributable to age dependent development of the MS.

Oktay analyzed 189 patients aged between 6 and 30 years, whereas Endo et al.^22^ included males and females of almost the same age. However, Oktay^1^ and Endo^22^ using bi-dimensional OPGs evaluated significantly distorted images of maxillary sinuses, with huge limitations in the methods which might well have hampered the interpretation of their findings, whilst the CBCT scans evaluated in this paper were significantly more accurate for MS three dimensional measurements.

Regarding linear measurements of the MS only LMW showed a statistically significant difference in ANB groups resulting larger in class I patients compared with class II and III (Table 3).

This is allegedly due to the association between class II and III malocclusion and maxillary hypoplasia that would reveal a reduced LMW. Various studies have reported that the anteroposterior and vertical problems related to Class II and III malocclusions are not the only related factors and that there is often an association with posterior transverse discrepancy^36–39^.

Class II and class III patients presented a reduced transversal dimension of the MS that had never been reported before in literature. This finding is strengthened by the Pearson’s correlation coefficients of the distance between the left and right Mx points with MSV, MSS and LMW that evidenced very high positive correlation (p-value < 0.01), as reported in Table 7.

Moreover, it was observed that females had a smaller LMW and LMH than males. No statistically significant interaction was noted for MS variables between gender and skeletal classes, with a similar trend for the MSV and MSS in the three SNA classes. This finding is inconsistent with those published by Oktay^1^ who evaluated the MS areas on OPGs of patients with different dental malocclusions defined according to Angle classification. Oktay^1^ found a significant interaction between gender and Angle’s class in the dimensions of the MS.

Differently from other previous research, the authors decided to make a 3D investigation assessing the influence that the cranio-maxillary relationship (SNA) may have on MS dimension. The authors supposed that the cranio-maxillary relationship (SNA) would have influenced maxillary sinuses size more than the sagittal skeletal jaw relationship (ANB).

The data of the present study showed that subjects with a retrusion (SNA < 80°) and protrusion (SNA > 84°) of the maxillary bone had a significant increase in MSV, MSS, LMW, LMH and LMD. Whilst there was no difference between genders for MSV, MSS and LMD. Conversely, males had larger LMW and LMH.

However, the data of the present study as to the relationship between SNA and MS dimensions cannot be compared to other research data as, to the best of authors’ knowledge, this is the first time this has been calculated. Interestingly, the evaluation of the significant interaction between the SNA classes and gender demonstrated a different tendency for the three linear variables between genders (Table 4).

The statistical interaction between SNA and LMW evidenced that females with a maxillary retrusion had smaller transversal sinus dimensions than males with the same condition. Conversely, males with maxillary protrusion (SNA > 84°) had smaller LMW than females.

The significant LMH findings on the interaction between SNA and gender evidenced that there was a different pattern for the two genders, i.e., in subjects with a reduced SNA, females presented smaller MS than males. A statistically significant interaction was found for LMD in subjects with normal SNA values. Whilst males presented significantly smaller LMD in subjects with normal SNA values compared with the other two subgroups, the female groups presented similar values for LMD in patients with normal or augmented SNA values and increased values of LMD in patients presenting reduced SNA values.

Several cephalometric measurements presented a positive correlation with the dimensions of the MS. These data allegedly sustain the hypothesis for the MS to be larger in patients presenting larger maxillofacial structures. Another study^32^ similarly reported that the MSV was positively correlated to the dimensions of the maxillofacial structures. This finding was in line with the data of the present study, even if Koppe et al.^31^ considered just three cephalometric parameters, that are the distance between the Mx points, facial height, and maxillary length.

The presented data did not evidenced any significant correlation between the SNA, SNB, and ANB angles and any MS size. This finding suggested that the anteroposterior dimension of the maxilla and of the mandible had neither a positive nor a negative correlation on MS size, although the ANB and SNA angles are both the criteria for the classification of the analyzed subjects (Table 7). These findings were in agreement with those previously published by Endo et al.^21^ who did not report any significant correlation between SNA, SNB and ANB angles and MS dimension. The abscence of a significant correlation between SNA and all the maxillary measurments is due to a V shape distribution of data between the SNA classes, with greater maxillary values in subjects with a retrusion and protusion of the maxillary bone.

Individuals with bigger S-N and PNS-A distances did tend to present larger MS. The correlation analysis of the MS measurements and the cranial base showed a positive correlation between S-N distance and MSV, MSS and LMD. Moreover, the significant positive correlation between the Ba-S-N angle and the LMD demonstrated that the angle increased along with the increase in depth. Conversely, the height and width were negatively correlated with the Ba-S-N angle. The data presented in this paper regarding Pearson’s correlation analysis between MS dimensions and PNS-A distance are in line with the one reported by Endo et al.^22^, that is subjects with longer nasomaxillary complexes have deeper and greater sinus, in terms of volume and surface. To understand this relationship we should consider that the MS, a pyramid-shaped sinus within the maxillary bone, is the biggest among paranasal sinuses^40,41^ as previously mentioned, contributing to midfacial growth and appearance^22,42,43^.

Endo et al.^22^ also reported a strong positive correlation between total maxillary sinus area (TMSA) and upper maxillary sinus area (UMSA) and S-N-Ans-Pns, showing a tendency for steeper maxillary planes to be positevely related with TMSA and UMSA. The results of the current research differ from those of Endo et al. as no relationship was observed between the inclination of the palatal plane (S-N/Ans-Pns) and the MS dimension and only the maximum height (LMH) had a statistically significant positive correlation with S-N-Ans-Pns.

The present correlation analysis between the vertical dimension and the MS measurements showed that patients with an augmented anterior vertical dimension should have larger MSV, MSS and greater LMH, and the subjects with a higher posterior facial height should have larger MSV, in accordance with data published by Endo et al.^22^. However, data comparison is not reliable as those previously published by Endo et al. were calculated on subjects aged from 12 to 16, a period when the maxillary size could be affected by a peak of growth.

Most of the studies are limited to the evaluation of the sinuses during orthodontic treatment or for a particular malocclusion and are impaired by bidimensional biases as well.

The present study aimed at obtaining information on the three-dimensional characteristics of maxillary sinuses amongst nasal breathing adults that had not been given any orthodontic treatment and evaluating the relationship that different cephalometric measurements may have on MSV and MSS, in both genders. However, this study does have some limitations, i.e., the relatively small sample size, even if it did suffice for inferential statistics consideration and the fact that all the subjects were Caucasian. Therefore, the conclusions may not be extrapolated to other ethnicities. Furthermore, the use of SNA and ANB are not completely reliable to investigate skeletal pattern. The literature teaches us that none of the sagittal parameters proposed so far can be considered totally reliable. It often happens that two parameters are conflicting or over or underestimate the actual skeletal discrepancy. The SNA and the ANB angle are parameters commonly used for evaluating the sagittal relationship between the maxilla and the cranial base and between the maxilla and the mandible respectively. The ANB angle would tend to decrease with increasing age^44,45^ and is influenced by the position of the nasion point: both on the sagittal plane and on the vertical plane^46–49^.

As reported by previous studies it seems that the growth of the MS in both genders overlaps with the pubertal growth spurt, and that its development starts between the age of 9 and 11 in females and later in males, between 12 and 14^16^. However, one strong point of this study is the average age of the selected sample, as it ensures a three-dimensional evaluation of the MS in an age range that is no longer subject to any significant volumetric changes. Moreover, the excellent intra-operator and inter-operator agreement (Table 7) demonstrated a very high reliability and repeatability of the presented method and a very small random error for volumetric and linear measurements in the assessment of MS size. The data of the present study are in line with various other authors who demonstrated the reliability and utility of CBCT scans in a three-dimensional assessment and reconstruction of the paranasal and maxillary sinus^16,17,50^.

The maxillary sinuses are of particular interest in dentistry due to their proximity to the area dentists work in. Therefore, thorough knowledge of MS anatomy is a must to avoid not only maxillofacial surgery complications, but also to make a presurgical evaluation for dental implant planning, graft size estimation for sinus lift procedures and infra-zigomatic mini-screw placement. Besides common dental procedure, the study of the MS is important in forensics to determine the gender if the whole body is not be available^51^. In case of orthodontic treatment that includes movements of the posterior teeth at the level of the maxillary sinus, the clinician should have a special care in patients with a large dimension of the maxillary sinus(as in case of an augmented vertical dimension). Literature reports that orthodontic space closure in missing posterior maxillary teeth through the sinus is arduous and it is recommended to use light force systems for a successful effect^52,53^. Moreover intrusion of teeth located in posterior area associated with root apices protruding into the maxillary sinus may be difficult and slow and it also need for very slight forces^54,55^.

The observations made in this study as to the orthodontic malocclusions seem to be in contrast with those put forward by Sassouni and Forrest^56^, i.e., that sinuses do not have a bearing on facial balance and malocclusions. A more thorough knowledge of three-dimensional MS dimension in different malocclusions may well be of help in treatment planning, as it has a common relationship with patients’ craniofacial features. However, all this considered and despite the efforts, the authors encourage these preliminary results be taken with caution as no clear-cut evidence is yet available in literature as to the influence cephalometric indices and/or malocclusive traits have on MS morphology. Moreover, even if present, statistical significance does not mean clinical relevance or the presence of cause/effect relationships between the variables. Indeed, statistical significance may have several confounding factors related to sample selection or sample pooling. Therefore, the authors urge the scientific community to carry out further studies on larger samples and to investigate further into any differences between populations to enrich and hopefully confirm the findings herein.

## Conclusions

In conclusion, this study demonstrated statistically significant larger volumes in males than in females. There was also a significant reduction in the transverse dimension of the MS in subjects with skeletal class II and III malocclusion. Subjects with a retrusion or protusion of the maxillary alveolar bone had larger MSV, MSS, LMW, LMH and LMD than subjects with a normal cranio-maxillary relationship. A significant interaction was observed for LMW, LMH and LMD measurements, demonstrating a different pattern between genders. A statistically significant high positive correlation was observed between S-N, Pns-A, S-Go, Mx-R/Mx-r and MS dimension. Whilst no significant correlation was noted between the MS measurements and anteroposterior skeletal classes (ANB) and the cranio-maxillary relationship (SNA).

## Data Availability

The data underlying this article will be shared on reasonable request to the corresponding author.
